# Cell Membrane Is Impaired, Accompanied by Enhanced Type III Secretion System Expression in *Yersinia pestis* Deficient in RovA Regulator

**DOI:** 10.1371/journal.pone.0012840

**Published:** 2010-09-17

**Authors:** Fengkun Yang, Yuehua Ke, Yafang Tan, Yujing Bi, Qinghai Shi, Huiying Yang, Jinfu Qiu, Xiaoyi Wang, Zhaobiao Guo, Hong Ling, Ruifu Yang, Zongmin Du

**Affiliations:** 1 Department of Parasitology, Harbin Medical University, Harbin, Heilongjiang, China; 2 Laboratory of Analytical Microbiology, State Key Laboratory of Pathogen and Biosecurity, Institute of Microbiology and Epidemiology, Academy of Military Medical Sciences, Beijing, China; University of Hyderabad, India

## Abstract

**Background:**

In the enteropathogenic *Yersinia* species, RovA regulates the expression of invasin, which is important for enteropathogenic pathogenesis but is inactivated in *Yersinia pestis*. Investigation of the RovA regulon in *Y. pestis* at 26°C has revealed that RovA is a global regulator that contributes to virulence in part by the direct regulation of *psaEFABC*. However, the regulatory roles of RovA in *Y. pestis* at 37°C, which allows most virulence factors in mammalian hosts to be expressed, are still poorly understood.

**Methodology/Principal Findings:**

The transcriptional profile of an in-frame *rovA* mutant of *Y. pestis* biovar Microtus strain 201 was analyzed under type III secretion system (T3SS) induction conditions using microarray techniques, and it was revealed that many cell-envelope and transport/binding proteins were differentially expressed in the Δ*rovA* mutant. Most noticeably, many of the T3SS genes, including operons encoding the translocon, needle and Yop (*Yersinia* outer protein) effectors, were significantly up-regulated. Analysis of Yop proteins confirmed that YopE and YopJ were also expressed in greater amounts in the mutant. However, electrophoresis mobility shift assay results demonstrated that the His-RovA protein could not bind to the promoter sequences of the T3SS genes, suggesting that an indirect regulatory mechanism is involved. Transmission electron microscopy analysis indicated that there are small loose electron dense particle-like structures that surround the outer membrane of the mutant cells. The bacterial membrane permeability to CFSE (carboxyfluorescein diacetate succinimidyl ester) was significantly decreased in the *ΔrovA* mutant compared to the wild-type strain. Taken together, these results revealed the improper construction and dysfunction of the membrane in the *ΔrovA* mutant.

**Conclusions/Significance:**

We demonstrated that the RovA regulator plays critical roles in the construction and functioning of the bacterial membrane, which sheds considerable light on the regulatory functions of RovA in antibiotic resistance and environmental adaptation. The expression of T3SS was upregulated in the *ΔrovA* mutant through an indirect regulatory mechanism, which is possibly related to the altered membrane construction in the mutant.

## Introduction

There are three *Yersinia* species that are pathogenic to humans. *Y. pseudotuberculosis* and *Y. enterocolitica* are enteric pathogens that generally cause self-limiting gastroenteritis or enterocolitis through consumption of contaminated food or water, whereas *Y. pestis* is the etiological agent of deadly plague, which is usually transmitted through the bites of infected fleas, direct contact with infected individuals or inhalation of infectious materials [1]. Although occurrence of a large-scale plague epidemic is minimally probable at present, small outbreaks in different countries have been reported to the World Health Organization every year [2], [3]. Because of high mortality rate and its potential transmission by inhaled aerosols, pneumonic *Y. pestis* represents a significant concern as an agent of bioterrorism [3].

RovA is a member of the MarR/SlyA family of global regulators, and proteins of this family are structurally conserved and are considered to be ubiquitous among bacteria [4]. Members of the MarR/SlyA family of proteins regulate a wide variety of functions including antibiotic resistance, environmental adaptation, production of antimicrobial agents and virulence factors. RovA analogous proteins in other pathogens, such as SlyA in *Salmonella typhimurium* and *Escherichia coli*, Rap in *Serratia marcescens* and AphA in *Vibrio cholerae*, have been implicated in the regulation of virulence. In human pathogenic *Yersinia* species, RovA has been shown to coordinate multiple metabolic, stress and virulence genes in response to environmental signals in the infected host [5]. *Yersinia* RovA was first identified as a regulator of invasion factor expression in a transposon mutagenesis screen of *Y. enterocolitica* using an *inv*::phoA reporter to monitor *inv* expression [5]. However, Inv, an important adhesion and invasion factor for the virulence of enteropathogenic *Yersinia* species, is inactive in *Y. pestis*. To address the role of RovA in pathogenesis of *Y. pestis*, Cathelyn *et al.* defined the RovA regulon in *Y. pestis* strain CO92, and they demonstrated that RovA is a global regulator that is indispensable for dissemination and colonization of the spleen and lungs in mice infected by the *s.c.* route and that it can directly bind to the promoters of *psaA* and *psaE* to contribute to the virulence of *Y. pestis* [6].

Expression of *rovA* has been shown to be subject to a positive auto-regulatory mechanism, and maximal *rovA* expression is achieved during stationary phase at 26°C, and much lower levels were detected at 37°C. A recent study indicated that RovA is an intrinsic temperature-sensing protein and that thermally-induced conformational changes in RovA interfere with its DNA-binding capacity and render it susceptible to proteolytic degradation. RovA can relieve the repression of the H-NS/YmoA complex by directly competing for binding to the promoters of the regulated genes including *inv* [7], [8], [9]. RovM, a LysR regulator implicated in the environmental control of virulence factors, has been demonstrated to negatively regulate *rovA* expression [10].

Pathogenic *Yersinia* species harbor a pathogenesis mechanism of type III secretion system (T3SS) that is required for virulence in mammals. It is encoded by a 70-kb plasmid shared by all three pathogenic species [11]. *Yersinia* T3SSs can deliver a group of Yop effectors into host cells through a secretion machine called the injectisome which spans the bacterial inner membrane, periplasm and outer membrane, leading to the inhibition of the host immune response [12]. The structure of T3SS is composed of two distinct parts. One is the hollow extracellular structure termed the ‘needle’ and the other is the cylindrical base that is embedded in the two bacterial membranes and ensures the stabilization of the T3SS structure on the cell envelope[13]. Regulation of the *Yersinia* T3SS involves complex mechanisms. LcrF/VirF, a regulator of the ArcA family [14], [15], is required for the activation of type III genes in response to the temperature shift from 26 to 37°C, which occurs during bacterial invasion into the mammalian host from arthropod vectors. YopN, SycN, LcrG, and TyeA have been shown to be involved in negative regulation of Yop secretion by acting cooperatively as a stop valve to block Yop secretion under secretion non-permissive conditions [16], [17], [18]. LcrQ is another negative regulator that inhibits the production and secretion of Yops in the bacterial cytoplasm, and it is rapidly secreted under secretion-permissive conditions, leading to Yop secretion [19]. YopD has been found to be associated with this inhibitory feedback mechanism, as the expression of Yop and LcrV was constitutively induced in the *yopD* mutant in the presence of calcium [20]. Cathelyn *et al.* used microarray analysis to define the RovA regulon in *Y. pestis* grown at 26°C in BHI, and their results revealed important regulatory roles of RovA in *Y. pestis*. During plague infection, the expression of various virulence mechanisms, including T3SS, are stimulated by an adverse host environment, and the mammalian body temperature of 37°C is one of the most important environmental signals sensed by this bacterium. Therefore, to clarify the regulatory roles of RovA on *Y. pestis* virulence under culture conditions mimicking the mammalian host environment is of great importance. In this study, transcriptional profiling of the *ΔrovA* mutant was analyzed under T3SS-inducing conditions using DNA microarray techniques. It was demonstrated that the transcription of many cell envelope and transport/binding proteins was actively regulated in the *ΔrovA* mutant, accompanied by significant enhancement of T3SS genes, such as operons encoding the translocon, needle and Yop effectors. Our results indicate that the RovA regulatory protein of *Y. pestis* is crucial for maintaining the bacterial membrane in a structurally and functionally normal condition, and upregulation of T3SS expression by RovA involves an indirect mechanism that will need to be determined in further studies.

## Results

### Construction and characterization of the *ΔrovA* mutant

**Figure 1:**
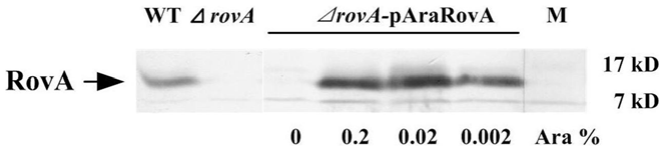
RovA expression in *Y. pestis* strain 201, the *ΔrovA* mutant and *ΔrovA-*pAraRovA. Overnight cultures of bacterial strains grown in BHI at 26°C were harvested and whole cell lysates were separated by SDS-PAGE. The expression of *rovA* was detected by Western blotting using a rabbit polyclonal antibody against His-tagged RovA. For the induction of RovA expression in *ΔrovA-*pAraRovA, arabinose was added to the culture medium at the indicated concentration.

An in-frame *rovA* deletion mutant of *Y. pestis* strain 201 with 41–139 aa deletion of RovA was constructed using the suicide vector-based method. The whole length of the *rovA* gene and the 600-bp sequence upstream of the *rovA* gene was cloned into pBAD24, generating pAraRovA, which was then used to complement the *ΔrovA* mutant. To confirm the successful construction of the *ΔrovA* mutant and the complementary strain, the parent strain, the *ΔrovA* mutant and *ΔrovA-*pAraRovA were grown in BHI broth at 26°C to stationary phase, when maximal *rovA* expression has previously been shown to be achieved [21]. The expression of RovA in each strain was determined by Western blot analysis (Figure 1). It was observed that a 17-kD band representing RovA could be detected in both the wild type strain and *ΔrovA-*pAraRovA, but not in the *ΔrovA* mutant, indicating that the *rovA* gene had been successfully disrupted. Introduction of exogenous expression of the *rovA* gene under the control of the arabinose-inducible promoter *araBADp* into the mutant strain restored RovA expression when induced with arabinose. These results demonstrated that the *ΔrovA* mutant and the complementary strain *ΔrovA-*pAraRovA were successfully constructed.

**Figure 2:**
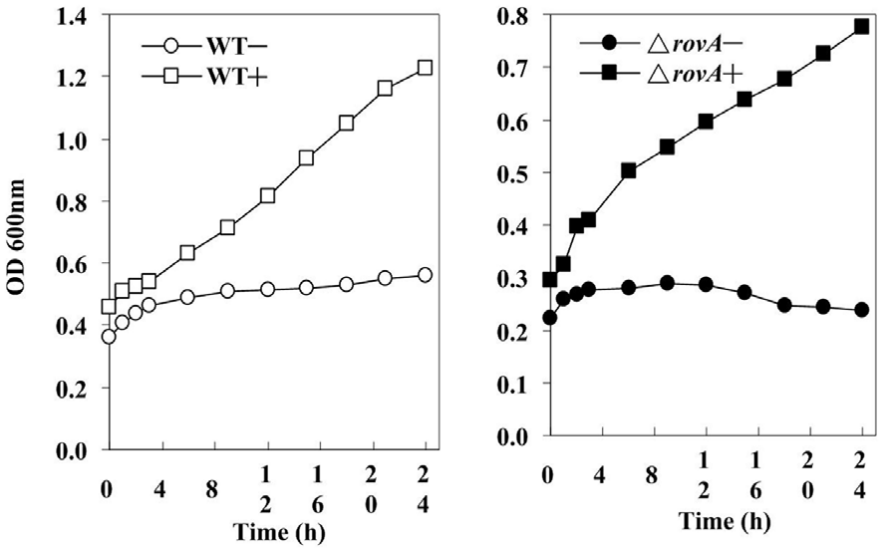
The low calcium response of the T3SS was not altered in the *ΔrovA* mutant. Overnight cultures of strain 201 and the *ΔrovA* mutant were diluted 20-fold into fresh TMH supplemented with 2.5 mM calcium (+) or not (−) and incubated at 26°C until the OD_600_ reached about 0.3, and then the cultures were transferred to 37°C. The OD_600_ value of the bacterial cultures was monitored at the indicated time points for 24 h until the cultures reached the stationary phase. Experiments were performed three times with similar results, and one representative result was shown.

Maximal *in vitro* expression of Yop and the T3SS machinery are reached at 37°C in the absence of millimolar concentrations of calcium and is generally accompanied by the growth restriction called the low calcium response (LCR) [11]. To investigate if mutation of the *rovA* gene affected the LCR, the *ΔrovA* mutant growth curves in TMH with and without 2.5 mM calcium were determined and compared with those of strain 201. Both the *ΔrovA* mutant and the wild type strain went into growth restriction when grown at 37°C in TMH without calcium, indicating that the *rovA* mutation did not affect the response of the T3SS to the low calcium environment (Figure 2). It was observed that the *ΔrovA* mutant grew slightly more slowly than the wild type strain in TMH with calcium, both at 26°C and 37°C (data not shown).

### T3SS genes were up-regulated in the *ΔrovA* mutant as revealed by microarray analysis

Comparison of the transcriptional profiles of strain 201 and the *ΔrovA* mutant grown in T3SS-inducing conditions revealed that the transcription of 141 genes was affected by the disruption of *rovA*. Among these, 69 genes were up-regulated and 72 were down-regulated compared to the wild type strain (See Supplementary Table S2 for details). These genes were distributed in 19 functional categories, according to the annotation of the *Y*. *pestis* CO92 genome (Supplementary Figure S1) [22], [23]. Interestingly, 19 of the type III secretion genes (over 40% of all the T3SS genes that have been already known) were differently expressed in the *ΔrovA* mutant and all of them were up-regulated, implying an enhancement of T3SS expression in the mutant. It was noticeable that 16 cell-envelope proteins and 21 transport/binding proteins were significantly affected by the mutation of *rovA* (Table 1), and these categories have a significantly higher number of affected genes than the other *rovA-*regulated gene categories (Supplementary Figure S1). The majority of the differentially regulated cell-envelope proteins were annotated as putative exported membrane proteins or lipoproteins with undefined function, while homologs of *wzzE, slyB, yggE, sbp1* and *yiaF* could be found in *E. coli*. Transport/binding proteins for amino acids, carbohydrates, organic acids, alcohols and ions were also actively regulated. These results implied that the structures and the transporting function of the bacterial membrane could be significantly affected in the mutant strain.

**Table 1:** RovA-regulated genes in the functional categories of cell envelope, transport/binding proteins and T3SS-encoded genes.

Gene ID	Fold change	Gene name	Product	Class number	Functional category
YPO0702	2.43	-----	putative exported protein	3.C.1	Cell envelop
YPO1689	1.93	-----	putative lipoprotein	3.C.1	Cell envelop
YPO1718	8.22	-----	putative exported protein	3.C.1	Cell envelop
YPO2373	7.69	*slyB, pcpY, pcp*	putative lipoprotein	3.C.1	Cell envelop
YPO2511	2.11	*-----*	putative exported protein	3.C.1	Cell envelop
YPO2782	2.93	*-----*	putative membrane protein	3.C.1	Cell envelop
YPO3865	2.46	*wzzE, wzz, b3785*	putative lipopolysaccharide biosynthesis protein	3.C.2	Cell envelop
YPO2477	2.37	*-----*	putative solute-binding protein	4.A	Transport/binding proteins
YPO1853	3.06	*putP*	proline permease	4.A.1	Transport/binding proteins
YPO2958	3.35	*sfuA, yfuA*	iron(III)-binding periplasmic protein	4.A.2	Transport/binding proteins
YPO0858	5.24	*-----*	sugar transport ATP-binding protein	4.A.3	Transport/binding proteins
YPO0860	2.28	*-----*	sugar-binding periplasmic protein	4.A.3	Transport/binding proteins
YPO0959	1.88	*-----*	putative sugar ABC transporter periplasmic binding protein	4.A.3	Transport/binding proteins
YPO1757	2.30	*manY, ptsP, pel*	PTS system, mannose-specific IIC component	4.A.3	Transport/binding proteins
YPO2501	2.51	*rbsB*	sugar binding protein precursor	4.A.3	Transport/binding proteins
YPCD1.05c	2.44	*sycE*	putative yopE chaperone sycE, yerA, yopE targeting protein		
YPCD1.16c	3.36		hypothetical protein		
YPCD1.17c	2.60	*ylpA*	putative lipoprotein precursor pseudogene, ylpA		
YPCD1.19c	4.15	*yopK, yopQ*	putative virulence determinant protein, yopK, yopQ		
YPCD1.20	2.69	*yopT*	putative cytotoxic effector protein, yopT		
YPCD1.21	2.52	*sycT*	putative yopT chaperone, sycT		
YPCD1.23	3.77		hypothetical protein, Y0062		
YPCD1.26c	8.09	*yopM*	probable targeted effector protein, yopM		
YPCD1.28c	3.16	*yopD*	putative Yop negative regulation/targeting component, yopD		
YPCD1.29c	2.79	*yopB*	putative Yop targeting protein, yopB		
YPCD1.30c	2.96	*lcrH, sycD*	putative yopB/yopD chaperone, lcrH, sycD		
YPCD1.31c	4.15	*lcrV*	putative V antigen, antihost protein/regulator, lcrV		
YPCD1.32c	3.43	*lcrG*	putative Yop regulator, lcrG		
YPCD1.37c	2.13	*sycN*	putative type III secretion protein, sycN		
YPCD1.49	2.02	*lcrF, virF*	putative thermoregulatory protein, lcrF, virF		
YPCD1.55	1.96	*yscF*	putative type III secretion protein, yscF		
YPCD1.60	1.94	*yscK*	putative type III secretion protein, yscK		
YPCD1.61	1.97	*yscL*	putative type III secretion protein, yscL		
YPCD1.71c	2.76	*yopP, yopJ*	putative targeted effector protein, yopP, yopJ		
YPO1222	–25.21	ompC, meoA, par	outer membrane protein C, porin	3.C	Cell envelop
YPO0063	–2.83	-----	putative membrane protein	3.C.1	Cell envelop
YPO0079	–4.64	*sbp1*	exported sulfate-binding protein	3.C.1	Cell envelop
YPO0917	–2.57	*yggE*	putative exported protein	3.C.1	Cell envelop
YPO2262	–3.82	*-----*	putative exported protein	3.C.1	Cell envelop
YPO2315	–3.34	*-----*	putative exported protein	3.C.1	Cell envelop
YPO2670	–4.12	*ureG*	urease accessory protein	3.C.1	Cell envelop
YPO4070	–3.16	*yiaF*	putative exported protein	3.C.1	Cell envelop
YPO2943	–2.78	*-----*	outer membrane usher protein (pseudogene)	3.C.3	Cell envelop
YPO2339	–2.08	*mppA*	putative periplasmic murein peptide-binding protein	4.A	Transport/binding proteins
YPO4110	–2.71	*-----*	ABC transporter permease	4.A	Transport/binding proteins
YPO4111	–3.77	*-----*	putative periplasmic solute-binding protein	4.A	Transport/binding proteins
YPO1937	–3.73	*ansP*	L-asparagine permease	4.A.1	Transport/binding proteins
YPO4109	–4.28	*-----*	putative amino acid transport system permease	4.A.1	Transport/binding proteins
YPO2338	–3.86	*-----*	CorA-like Mg2+ transporter protein	4.A.2	Transport/binding proteins
YPO0182	–4.69	*tauA, ssiA*	putative taurine-binding periplasmic protein precursor	4.A.3	Transport/binding proteins
YPO0183	–2.32	*tauB, ssiB*	putative taurine transport ATP-binding protein	4.A.3	Transport/binding proteins
YPO0184	–6.41	*tauC, ssiC*	putative taurine transport system permease protein	4.A.3	Transport/binding proteins
YPO3012	–2.83	*cysA*	sulfate transport ATP-binding protein	4.A.5	Transport/binding proteins
YPO3014	–2.94	*cysT*	sulfate transport system permease protein CysT	4.A.5	Transport/binding proteins
YPO3015	–2.84	*cysP*	thiosulfate-binding protein	4.A.5	Transport/binding proteins
YPO3624	−5.91	*ssuA*	putative aliphatic sulfonates binding protein	4.A.6	Transport/binding proteins

### The *ΔrovA* mutant was slightly attenuated in the mouse model after *s.c.* and *i.v.* infections

**Figure 3:**
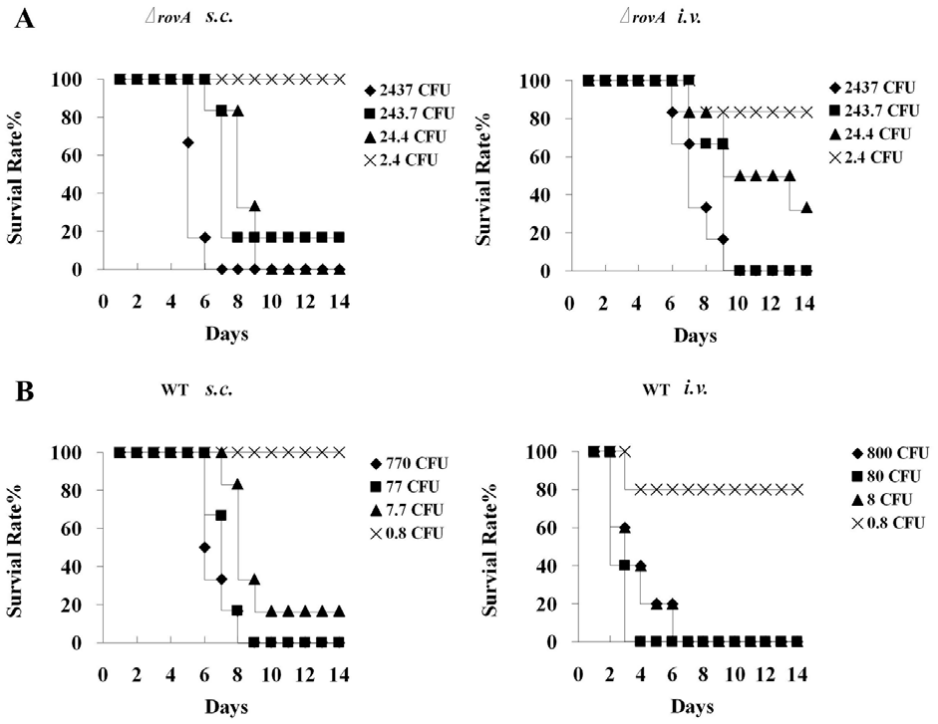
Virulence analysis of *Y. pestis* strain 201 and the Δ*rovA* mutant in a mouse model. BALB/c mice were challenged with bacterial suspensions of the *ΔrovA* mutant (A) or strain 201 (B) in PBS at the indicated concentrations via the *s.c.* or *i.v*. routes of infection, and the survival rates were plotted against the days post infection.

To evaluate the contribution of RovA to the virulence of strain 201, half lethal dose (LD_50_) analysis was conducted with the wild type strain and the *ΔrovA* mutant in BALB/c mice. A previous study by Cathelyn *et al.* demonstrated that RovA is required for full virulence of strain CO92, and a *ΔrovA* mutant was attenuated 80-fold based on LD_50_ analysis after *s.c.* inoculation, however, it was only slightly attenuated in *i.n.-* or *i.p.-*infected mice [6]. In contrast to the results of Cathelyn *et al*, we found that more significant virulence attenuation of the *ΔrovA* mutant was observed in the *i.v.*-infected mice than in *s.c.-*infected mice, although the attenuation was slight in both routes of infection. When mice were infected by *s.c.* injection, the LD_50_ values for strain 201 and the *ΔrovA* mutant were 3 and 9.2 cfu, respectively (Figure 3), while the LD_50_ in the *i.v.*-infected mice were 1.9 and 10.5 cfu for the wild type and the *ΔrovA* mutant strain, respectively. The mean day to death was delayed in both *i.v.-* and *s.c.*-infected mice, with a more significant delay in *i.v.* infection (Figure 3). This disagreement between our results and those of Cathelyn *et al.* possibly result from the distinct experimental conditions employed, from the animals used to the bacterial growing conditions. More importantly, the wild type *Y. pestis* strains used in the two studies were different from each other, i.e., the biovar microtus strain 201 was used in this study while biovar orientalis strain CO92 was used by Cathelyn *et al*.

### T3SS is up-regulated in the *ΔrovA* mutant at both the transcription and expression levels

**Figure 4:**
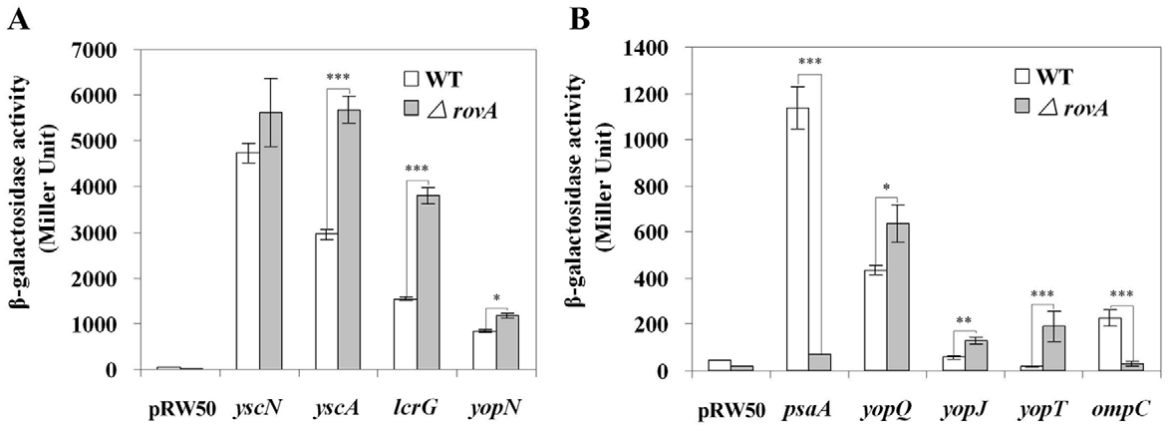
Effect of the RovA protein on the promoter activities of the regulated loci. *Y. pestis* strain 201 and the *ΔrovA* mutant harboring empty vector pRW50 or pRW50 derivatives containing the corresponding promoters were grown in TMH without calcium at 26°C to an OD_600_ of 1.0, and then transferred to 37°C for of 3 more hours of incubation. β-Galactosidase activities in Miller units were shown for promoter activities of *yscN, yscA, lcrG* and *yopN* (A), and for promoter activities of *psaA, yopQ, yopJ, yopT* and *ompC* (B). Data shown are the mean values of three independent experiments with the standard deviations indicated. The difference of β-galactosidase activities between the wild type strain and the *ΔrovA* mutant were calculated using Student's t test, and a *p* value of <0.05 was taken as statistically significant. The *p* value is indicated as follows: *p*<0.05 *, *p*<0.01 **, *p*<0.001 ***.

To validate the regulatory role of RovA on the transcriptional activities of T3SS genes, we constructed serial pRW50 derivatives containing *lacZ*-fused promoters (Table 2). The gene organization of the *Yersinia* T3SS consists of four large transcriptional units, including the *yopNtyeAsycNyscXYLcrDR, yscABCDEFGHIJKLM, yscNOPQRSTU* and *lcrGVHYopBD* operons, the dispersed genes in plasmid pCD1 including *virG/yscW*, *yops* and their chaperone *syc* genes. Therefore, we selectively constructed the *pyopN*-, *pyscA-, pyscN-* and *plcrG-lacZ* fusions to represent the transcriptional activities of those operons in different background strains. Plasmids containing *yop*-promoters (p*yopT*, p*yopJ*, p*yopQ*) directly upstream of the *lacZ* gene expressed much higher levels of β-galactosidase in the *ΔrovA* mutant compared to the wild type strain (Figure 4B). Among the four large operons that encode distinct parts of T3SS machine, *lcrGVHyopBD* and *yscABCDEFGHIJKLM* were found to be upregulated in the *ΔrovA* mutant in transcriptional profiling analysis (>2-fold), while for the other two operons, *yopNtyeAsycNyscXYLcrDR* and *yscNOPQRSTU*, no significance differences were detected. In good accordance with the microarray results, the β-galactosidase activities expressed by p*lcrG* and p*yscA* were significant higher in the *ΔrovA* mutant than in the wild type strain, but only a limited difference was observed for p*yopN* (far below 2-fold) and no difference was observed for p*yscN* (Figure 4A). The *psaA* gene has been shown to be positively regulated by RovA [6], and *ompC*, which has been previously shown to be associated with membrane permeability, was down-regulated by >20 fold in the *ΔrovA* mutant according to the microarray analysis. Therefore, the two genes were also included in the β-galactosidase assays, and the results validated the microarray analysis results showing that *pasA* and *ompC* were significantly down-regulated in the *ΔrovA* mutant. In summary, β-galactosidase activity assays demonstrated that mutation of the *rovA* gene significantly elevated the expression of T3SS components, which confirmed the microarray results.

**Table 2:** Bacterial strains and plasmids used in this study.

Strain or plasmid	Relevant characteristics	Source or reference
DH5α	Cloning host	Laboratory collection
SPY372*λpir*	Host for replication of suicide vector pGMB151	From Prof. Huang X. [40]
strain 201	*Y. pestis* biovar. microtus	[23]
*ΔrovA*	strain 201, *ΔrovA*(41–139aa)	This study
*ΔrovA-*pAraRovA	the *ΔrovA* containing pAraRovA, Ap^r^	This study
pRW50	Low-copy-number lac exprssion reporter vector, Tet^r^	From Prof. Green J. [45]
pBAD24	*araBADp* cloning vector, Ap^r^	Laboratory collection
pAraRovA	pBAD24 containing rovA gene at *Eco*RI and *Sma*I	This study
pGMB151	suicide vector; SacB1 R6K origin; Sm^r^	From Prof. Huang X.[40]
pGMB-del*rovA*	pGMB151 containing ∼700-bp sequences up- and downstream of the *rovA* gene	This study
p*yscN*	538-bp *yscN* promoter inserted at *Eco*RI-*Hin*dIII sites, Tet^r^	This study
p*yscA*	496-bp *yscA* promoter inserted at *Eco*RI-*Hin*dIII sites, Tet^r^	This study
p*lcrG*	513-bp *lcrG* promoter inserted at *Eco*RI-*Bam*HI sites, Tet^r^	This study
p*yopN*	567-bp *yopN* promoter inserted at *Eco*RI-*Hin*dIII sites, Tet^r^	This study
p*yopT*	772-bp *yopT* promoter inserted at *Eco*RI-*Hin*dIII sites, Tet^r^	This study
p*yopQ*	578-bp *yopQ* promoter inserted at *Eco*RI-*Hin*dIII sites, Tet^r^	This study
p*yopJ*	502-bp *yopJ* promoter inserted at *Eco*RI-*Hin*dIII sites, Tet^r^	This study
p*psaA*	538-bp *yscA* promoter inserted at *Eco*RI-*Hin*dIII sites, Tet^r^	This study
p*ompC*	509-bp *ompC* promoter inserted at *Eco*RI-*Hin*dIII sites, Tet^r^	This study

**Figure 5:**
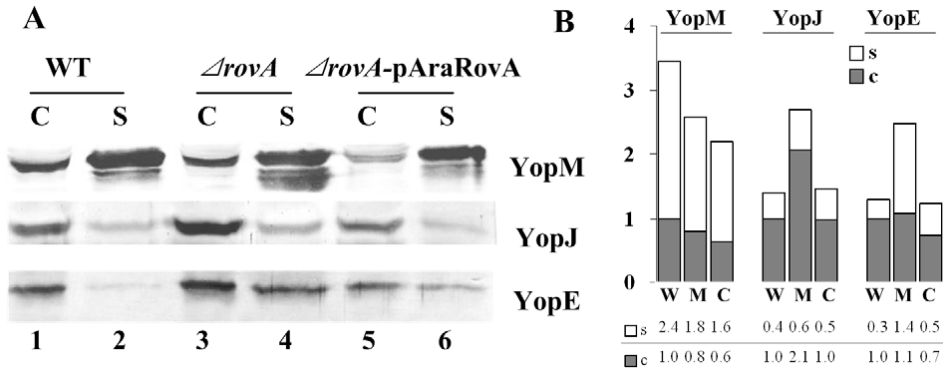
Influence of RovA on Yop expression and secretion. Bacterial strains were grown in TMH medium without calcium at 26°C to an OD_600_ of ∼1.0 and then transferred to 37°C for 3 h to induce the expression and secretion of Yop proteins. TCA was used to precipitate proteins from the culture supernatants. The bacterial cell pellets were separated by SDS-PAGE, and specific proteins were detected using rabbit polyclonal antibodies against YopE, YopJ and YopM (A). C and S stand for proteins separated from the cell pellet and bacterial culture supernatant, respectively. In lane 4, the band right below the YopM band could be the degraded product of YopM, and a large amount of this product could be stably detected in the *ΔrovA* mutant; however, it was much less in the wild type and the *ΔrovA* mutant complemented with *ΔrovA-*pAraRovA. Densitometry analysis of Western blots was performed using TotalLab software, and the numbers indicate the ratios of the densitometry values from each lane to lane 1 of each row (B). W: wild type strain; M: mutant strain *ΔrovA*; C: complementary *ΔrovA-*pAraRovA strain.

To determine whether Yop expression by the T3SS also increased in the *ΔrovA* mutant as well, the Yop proteins secreted from the culture supernatant and the cell pellets of bacterial cultures grown in TMH without calcium at 37°C were analyzed by SDS-PAGE separation and Western blotting. Yop secretion and expression assays for distinct Yop variants were performed at least three times, and it could be consistently observed that expressions of YopE and YopJ were higher in the *ΔrovA* mutant than in the wild type strain (Figure 5, comparing lanes 3 and 4 to lanes 1 and 2). The amount of secreted YopE and YopJ in the culture supernatant also increased significantly compared to the wild type strain (Figure 5, comparing lanes 2 and 4). However, no significant difference could be detected in YopM expression. A strong band just below the YopM band could be detected repeatedly in several independent experiments (lane 4), and we assumed that it could be a degraded peptide from YopM. If the degraded products of YopM are taken into account, the expression of YopM in the *ΔrovA* mutant is almost equal to that in the wild type strain. Yop expression and secretion were restored partially by introducing the trans-complemented *rovA* gene into the *ΔrovA* mutant (Figure 5, lane 5 and lane 6). These data suggested that type III secretion genes were up-regulated at both the transcriptional and expression level.

### RovA cannot bind to the prompters of *rovA*-regulated T3SS genes

**Figure 6:**
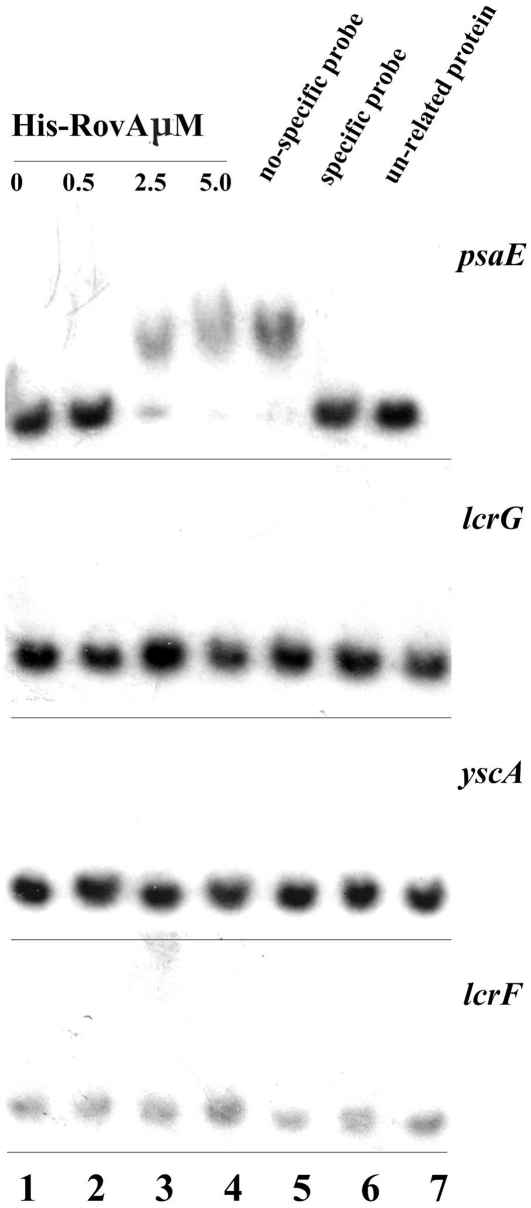
Ability of RovA to bind to promoters of *lcrF* and *lcrG* of T3SS. The upstream regions of *lcrG, yscA, lcrF* and *psaE* were amplified by PCR and used as target DNA probes in EMSA. The [γ-32P]-labeled target DNA probes were incubated with or without increasing amounts of purified His-RovA protein (lanes 1–4). Three controls were included in each EMSA experiment as indicated: 1) non-specific probe competitor (unlabeled DNA probe containing promoter of a gene that was shown to be not affected by *rovA* mutation); 2) specific probe competitor (unlabeled DNA probe containing promoter region of the investigated gene); and 3) unrelated proteins (rabbit anti-F1-protein polyclonal antibody).

To determine whether this up-regulation of T3SS genes was implemented via a direct or indirect mechanism, electrophoresis mobility shift assays (EMSA) were carried out. Transcription of the *lcrGVHyopBD* and *yscABCDEFGHIJKLM* operons was up-regulated in the *ΔrovA* mutant as revealed by microarray analysis, and therefore, RovA was tested for its ability to bind to the promoter regions of *lcrG* and *yscA*. The transcriptional activator LcrF is required for the induction of T3SS transcription in response to an environmental temperature shift from 26 to 37°C. To see whether RovA can control the transcription of T3SS through direct regulation on *lcrF*, the binding activity of RovA to the promoter of *lcrF* was also determined. *psaEFABC* has been shown to be directly regulated by RovA, and the promoter sequence of *psaE* was able to bind with the RovA protein. Therefore, the *psaE* gene was included in the experiment as a positive control. The results showed that although the retardation of the promoter sequence of *psaE* by RovA could be consistently detected (Figure 6), we were unable to observe binding activity of RovA to the promoter sequences of *lcrF*, *lcrG* and *yscA* (Figure 6). These results suggested that RovA could regulate the expression of T3SS via an indirect mechanism.

### Characterization of the cell membrane of the *ΔrovA* mutant by transmission electron microscopy

**Figure 7:**
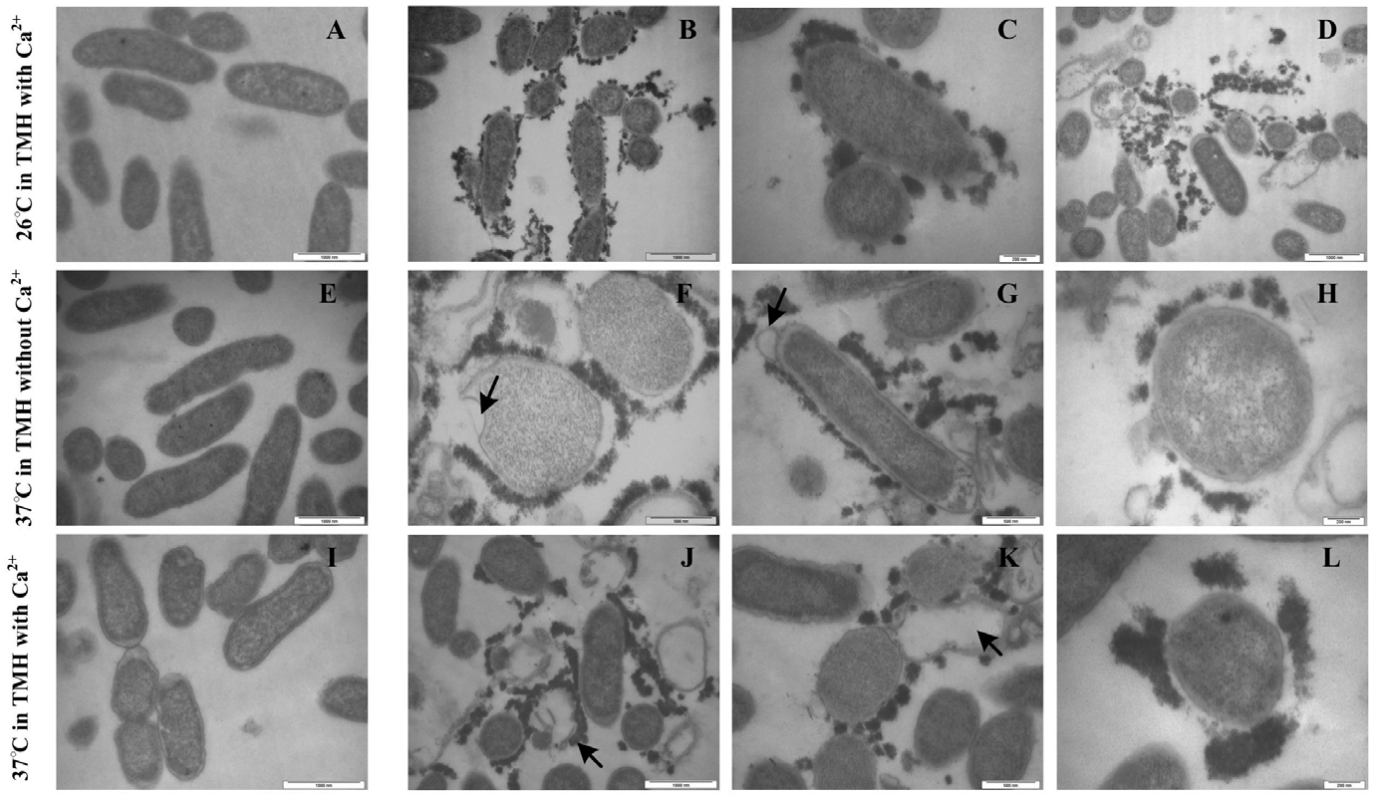
RovA mutation leads to alteration of the bacterial membrane. Bacterial strains were either grown in TMH medium with 2.5 mM calcium at 26°C to the early stage of the stationary phase (A, wild type strain; B, C and D, *ΔrovA* mutant) or grown in the same medium to an OD_600_ of 0.3 and then transferred to 37°C for 3 h (E, wild type strain; F, G and H, *ΔrovA* mutant); or grown in TMH without calcium at 26°C to an OD_600_ of 0.3 and then transferred to 37°C for 3 h (I, wild type strain; J, K and L, *ΔrovA* mutant). Bacterial cells were harvested and subjected to transmission electron microscopy observation. Bacterial cells of the *ΔrovA* mutant were shown to be surrounded by electron dense particles in B, C, F, G, H, K and L. Arrows in J and K indicate the disrupted cells, and the covering of electron dense materials around the disrupted cells could be clearly observed. Arrows in F and G indicate bubbles on the bacterial membrane. Bars indicate 200 nm in C, H and L; 500 nm in F, G and K; 1,000 nm in A, B, D, E, I and J.

The wide regulatory influence of RovA on cell envelope proteins and proteins involved in cell transport/binding processes suggested that cell membrane structure and function might be impaired in the *ΔrovA* mutant. This prompted us to visualize the bacterial cell membranes using transmission electron microscopy (TEM). When grown in TMH at 26°C, TEM results illustrated that the bacterial membranes of the wild type strain were smooth and the boundaries were sharp and clear (Figure 7A). However, for many of the *ΔrovA* mutant cells, many small particle-like structures with high electron density were found to surround the membranes (Figure 7B, C, D), and aggregated large clumps that were possibly formed from the small particle-like structures were frequently found near the disrupted cells (Figure 7D). A noticeable fraction of the *ΔrovA* mutant cells appeared to be lysed, suggesting that the bacterial membranes might be constructed differently, thus unstable in the *ΔrovA* mutant under the experimental conditions employed, leading to the disruption of the bacterial cells. By counting the wild-type bacterial cells that were surrounded by the electron-dense particles and those that were either lysed or apparently healthy from ten randomly selected visual fields, we found that no lysed bacterial cells could be observed, and approximately 2% of cells were shown to be associated with electron-dense particles. However, these numbers for the *ΔrovA* mutant were 11% and 28%, respectively. Mammalian host body temperature is 37°C, which will greatly increase the transcription of the T3SS through the action of the thermo-regulator LcrF, and the lack of a mill molar concentration of calcium in the environment at 37°C will trigger the secretion of Yop proteins and fully activate the transcription of the T3SS. Therefore, we conduct TEM analysis to determine the impact of RovA on the bacterial membrane under T3SS transcription and secretion induction conditions. When the *ΔrovA* mutant cells were grown at 37°C in TMH with and without calcium, similar results were observed as described for bacteria grown at 26°C, such that many *ΔrovA* mutant cells were surrounded by electron-dense particles, and some of the cells were damaged to different degrees (Figure 7 F–H, J–L). Bubbles could be observed at the surface of some *rovA* mutant cells (Figure 7 G and F), and some cells were totally lysed and surrounded by remaining electron-dense materials (Figure 7 J). Together, these results indicated that cell membrane construction and cell integrity in *Y. pestis* showed significant dependence on RovA whether the bacteria were grown under T3SS-inducing or non-inducing conditions, which suggests that RovA could play a critical regulatory role in the construction of the cell membrane and is necessary for the maintenance of the stability of the cell membrane.

### Membrane permeability of the *ΔrovA* mutant is significantly decreased

**Figure 8:**
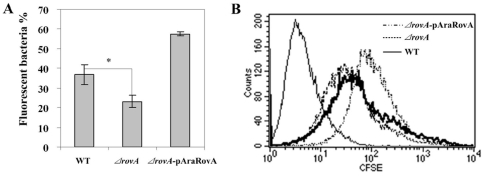
Cell membrane permeability to CFSE is lowered in the *ΔrovA* mutant. Strain 201, the *ΔrovA* mutant and *ΔrovA-*pAraRovA were incubated with 1 µM CFSE for 10 min and subjected to flow cytometry analysis. The percentages of fluorescent bacteria are shown as means ± standard deviation of three replicate experiments (A). Bacteria stained by CFSE for wild type (solid line), *ΔrovA* mutant (dotted line) and *ΔrovA-*pAraRovA (dashed line) were shown by histograms. Data are representative of 3 independent experiments. The difference between the wild type and the *ΔrovA* mutant were calculated using Student's t test and *p*<0.05 (*p* = 0.02, indicated as *) was taken as statistically significant.

The cell membrane structure in the *ΔrovA* mutant has been shown to be disrupted by the *rovA* mutation by TEM. In order to examine the regulatory role of RovA on cell membrane functions, we next determined the membrane permeability of the *ΔrovA* mutant strain. CFSE (carboxyfluorescein diacetate succinimidyl ester) is a small molecule dye that can passively diffuse into the live cell. It is colorless and non-fluorescent, but once inside the cell, intense fluorescence can be produced by the action of intracellular esterases on the acetate groups and then be analyzed by flow cytometry [24], [25]. After the wild type and *ΔrovA* mutant cells were labeled by incubation with 1 µM CFSE in PBS at 37°C, the cells were washed thoroughly, resuspended in PBS buffer and subjected to flow cytometry analysis. The results demonstrated that the passive permeation rate of CFSE for the *ΔrovA* mutant was significantly decreased compared to that of the wild type strain, suggesting lowered membrane permeability in the *ΔrovA* mutant (Figure 8). When a *ΔrovA* mutant strain complemented with pAraRovA was subjected to CFSE staining and flow cytometry analysis, the percentage of stained cells was restored and was even higher than that of the wild type strain. This was possibly due to the overexpression of RovA under the control of the arabinose-inducible promoter *araBADp*, which was higher than the expression level in strain 201. *ompC* was observed to be greatly down-regulated in the *ΔrovA* mutant in this study, as described in the previous sections. OmpC is one of the major outer-membrane porins that are thought to influence permeability of the outer membrane, and reduced membrane permeability has been observed in an *ompC* mutant of *Y. enterocolitica* [26]. The down-regulation of OmpC expression, together with the active regulation of many cell envelope and transport/binding proteins by RovA could be responsible for the alteration of membrane permeability in the *ΔrovA* mutant. These data indicated that not only the cell membrane construction but also its functions were disrupted by mutation of the RovA regulator.

## Discussion

Virulence gene expression of *Y. pestis* is tightly regulated by environment signals, including temperature, nutrients, growth phase, osmolarity and pH. The typical temperature of arthropod vectors is about 26°C, whereas it is 37°C in rodents and temperature is thus an important signal for *Y. pestis* to sense stress environments in mammalian hosts [27], [28]. A temperature shift from 26°C to 37°C results in maximal expression of most of the virulence factors, including pH 6 antigen, F1 antigen, Pla protease and the T3SS [29], [30], [31]. In this study, we analyzed the transcriptional profile of the *ΔrovA* mutant under T3SS-inducing conditions, which allows the expression of most *Yersinia* virulence factors that are required in mammalian hosts, and we found that the expression of 19 T3SS-encoded genes was enhanced in a *ΔrovA* mutant. The gene function categories of cell envelope and transport/binding proteins, according to the annotation of CO92 genome, were the most significantly affected categories. There are few overlaps between the RovA regulons of this study and that was previously reported by Cathelyn *et al.* [6] and we believe that the great discrepancies may be the result of two fundamental causes. First, the bacteria were grown in different experimental conditions. In a study by Cathelyn *et al.*, bacteria were grown at 26°C in BHI medium, while in this study, bacteria were grown at 37°C in chemically-defined TMH medium without calcium, which is a condition that induces the expression of the T3SS and many other virulence factors required for successful infection of mammals. It could be highly possible that the RovA regulons are distinct in the bacteria grown at 26 and 37°C because the expression of RovA itself is thermo-dependent, and that the maximal expression of RovA occurs when the bacteria are grown to the early stage of stationary phase at 26°C [21], [32]. Second, the *Y. pestis* strains used in the two studies are different. Cathelyn *et al.* used the biovar Orientalis strain CO92, whereas in this study, we used biovar Microtous strain 201[23], [33], which is avirulent in humans, but highly lethal for mice. Previous reports have demonstrated that although the *rovA* sequence is identical in the three human pathogenic *Yersinia* species, the RovA regulons of *Y. enterocolitica* and *Y. pestis* are distinct [38]. This led to the suggestion that RovA regulates genetic materials that were horizontally transferred after the divergence of the species because a number of RovA-regulated loci in *Y. enterocolitica* do not have orthologs in *Y. pestis*, and vice-versa [34]. It could be possible that there is intrinsic variance between the RovA regulons of strains 201 and CO92.

We further confirmed the up-regulation of some *yop* genes, including YopT, YopQ and YopJ, and two large operons, *lcrGVHyopBD* and *yscABCDEFGHIJKLM*, of the T3SS by analyzing the β-galactosidase activity in serial *lacZ* fusions containing promoter sequences upstream of the corresponding genes. The *Yersinia* effectors YopE, YopM and YopJ were analyzed from bacterial cultures grown under T3SS-inducing conditions, and it was found that the expression of YopE and YopJ in the *ΔrovA* mutant was significantly higher than in the wild-type strain, while it appears that YopM was equally expressed in the wild type strain and the *ΔrovA* mutant. However, none of these experimental data can illustrate whether the regulatory role of RovA on the T3SS involves a direct or indirect mechanism. We performed EMSA experiments using a purified His-tagged RovA protein and PCR products containing the DNA sequence upstream of the *lcrF* gene, the *yscABCDEFGHIJKLM* and *lcrGVHyopBD* operons and the *psaEFABC* promoter, which has been shown to directly interact with RovA [6]. Although we could stably detect inhibition of the mobility of the *psaE* promoter sequence by RovA, no inhibition was observed for the promoters of *lcrG*, *yscA* and *lcrF*. These results strongly implied that the regulatory role of RovA in the T3SS is implemented via an indirect mechanism. *lcrF* was shown to be expressed at ∼2-fold higher levels in the *ΔrovA* mutant compared to the wild type strain based on DNA microarray analysis (Supplementary Table S2), and this upregulation should contribute, at least partially, to the increased expression of T3SS genes in the *ΔrovA* mutant. Heroven *et al.* analyzed the mechanism of RovA-dependent transcription of the *inv* gene and *rovA* itself [9]. They showed that RovA could bind to a high AT abundance region with a high occurrence of poly(AT) stretches, and they did not find a strong characteristic of palindromic sequences in the *inv* and *rovA* promoters recognized by RovA. Many members of the AraC family regulators have been shown to commonly target AT-rich tracts of the regulated genes [7], [9]. Until now, the molecular mechanism of RovA-dependent transcription of the target genes has only been determined for two genes, *inv* and *rovA*, and the analysis of more target sequences will obviously be required to fully characterize a RovA binding site. It has been shown that the binding sites of RovA overlap with H-NS-protected sequences in the regulated regions, and RovA could play its regulatory role through antagonizing H-NS-mediated silencing of *inv* and *rovA* expression in *Y. pseudotuberculosis* [7], [9].

We demonstrated by DNA microarray analysis that the categories of cell envelope and transport/binding proteins are the most actively regulated gene categories in the *ΔrovA* mutant, and this was validated by subsequent experiments showing that the cell membranes of the mutant bacteria were both structurally and functionally disrupted. Visualization of the *rovA* mutant cells by TEM analysis indicated that there were many small electron dense particles surrounding the mutant cells, and some cells had been lysed and electron dense material remained around a number of these, suggesting that the construction of the bacterial membrane might have been severely affected when the bacteria were grown both at 26°C and 37°C under Yop secretion-inducing or non-inducing conditions. These results are, again, in agreement with our transcriptome profiling of the *ΔrovA* mutant and demonstrated that the cell membrane is inadequately constructed in this mutant.

One of the most important functions of the cell membrane is to control cell permeability to environmental substances to maintain what we observed in homeostasis in the bacterial cytosol and to provide protection against adverse environmental factors. The decreased membrane permeability the *ΔrovA* mutant indicates that the bacterial membrane was also functionally disrupted in these cells. OmpC has been implicated in the permeability of cell membranes, and the decreased permeability of the mutant bacteria was in accordance with the observation in this study that the *ompC* gene was greatly downregulated.

The T3SS can assemble syringe-like structures of over 20 proteins, called injectisomes, spanning the two layers of the bacterial membrane [35], [36], whereby the effector proteins can be delivered into host cells. A recently published study by Cornelis *et al*. showed that injectisome assembly is initiated by the formation of the O-ring (outer membrane ring) in the outer membrane, and YscD is subsequently attached to YscC, followed by attachment of a lipoprotein, YscJ, which completes the formation of the MS ring (the lower ring spanning the inner membrane)[41]. Subsequently, the ATPase complex, consisting of YscN, K, L, and Q, assembles at the cytoplasmic side of the injectisome. Finally, needles composed of YscF and LcrV are formed. Based on the process of injectisome formation, we conjecture that alterations of membrane construction might interfere with these sequential procedures that occur at the bacterial outer and inner membranes. The membrane structure in the *ΔrovA* mutant might be more favorable for the insertion of YscC and YscD into the membrane, or might enhance the stability of the ring structures through an unknown mechanism, thereby promoting the assembly of injectisome. The secretion function of the T3SS could also be affected when membrane construction is altered. A feedback regulatory mechanism mediated by LcrQ is known to be involved in T3SS regulation and inhibits Yops secretion and synthesis under T3SS non-inducing conditions. It has been shown that functional secretion machinery is required for full expression and secretion of Yops through this feedback regulatory mechanism [11], [37]. The disordered membrane construction might affect the balance of the feedback regulation, resulting in significant enhancement of the transcription and expression of the T3SS. However, these assumptions needed to be further confirmed to clarify the linkage between membrane construction and assembly and function of the T3SS.

Investigations of the transcriptome and the proteome of *S. typhimurium slyA* mutants have identified many loci with altered expression in *slyA* mutant strains [38], [39]. Although the results obtained using two different approaches were different, the majority of the *slyA*-dependent genes are predicted to encode membrane, periplasmic or secreted proteins. These observations are quite similar to our results showing that a *rovA* mutation in *Y. pestis* results in differential expression in the gene categories of cell envelope and transport/binding proteins, in addition to enhanced expression of the T3SS. This suggests that the regulatory roles of MarR/SlyA family regulators in the cell membrane might greatly contribute to their extensive regulatory functions as global regulators of antibiotic resistance, environmental adaptation and production of antimicrobial agents and virulence factors.

In conclusion, we showed that RovA plays critical regulatory roles in the construction and function of bacterial cell membranes and is necessary to maintain the membrane in a structurally and functionally normal condition. The observed bacterial membrane alteration was accompanied by the enhancement of T3SS expression in the *ΔrovA* mutant through an uncharacterized indirect mechanism. This indicated to us that the disordered membrane structure might interfere with the functions of T3SS injectisomes spanning two layers of the cell membrane. These results suggest that other virulence factors that are exported at the bacterial surface or embedded in the membrane might be influenced as well, including *inv* and *psaA*, which have already been shown to be directly regulated by RovA.

## Materials and Methods

### Bacterial strains, plasmids and growth conditions

The bacterial strains and plasmids used in this study are listed in Table 2. *Y. pestis* biovar microtus strain 201 is avirulent in humans but highly virulent in mice. Bacterial strains were grown in Luria- Bertani (LB) broth, Brain-Heart Infusion broth (BHI) or chemically defined TMH medium with or without a final concentration of 2.5 mM calcium. Antibiotics were added to culture media when needed at the following concentrations: 100 µg/ml ampicillin, 20 µg/ml streptomycin, 10 µg/ml tetracycline and 20 µg/ml kanamycin.

### Mutagenesis and Plasmid Construction

The *ΔrovA* mutant was constructed using the suicide vector-based allelic exchange method [40]. The ∼700-bp sequences upstream and downstream of the *rovA* gene were PCR amplified using primers rovA-up-F/-R and rovA-down-F/-R, respectively (all primer sequences are listed in Table S1 as supporting information). The products were then digested using *Eco*RI and *Xho*I for the upstream fragment of *rovA*, and *Xho*I and *Sal*I for the downstream fragment of *rovA*, respectively. The two fragments were ligated and cloned into suicide vector pGMB151 in *E. coli* SPY372*λpir*, generating plasmid pGMB-delrovA. This plasmid was then electroporated into strain 201 and the recombinants were selected on LB plates with streptomycin and confirmed by PCR using primers rovA-F and rovA-R. The *ΔrovA* mutants losing the backbone of the suicide vector were counter-selected on LB plates with 5% sucrose. For construction of the complementary strain, a fragment containing the ∼500-bp sequence upstream of *rovA* and the *rovA* open reading frame was amplified using template DNA from strain 201. The PCR amplicon was doubly digested by *Sac*I and *Pst*I and then cloned into pBAD24, generating plasmid pAraRovA. After verification by DNA sequencing, the plasmid pAraRovA was subsequently introduced into the *ΔrovA* mutant to gain the complementary strain *ΔrovA-*pAraRovA.

PCR primers were designed for amplification of ∼500- to 600-bp sequences upstream of *yscA, lcrG, yscN, yopN, psaA, yopQ, yopJ, yopT* and *ompC* genes, which represent promoters of the corresponding downstream genes, according to the annotation of plasmid pCD1 of *Y. pestis* CO92. After PCR amplification using the template DNA from 201, the DNA fragments were digested and ligated into pRW50 just upstream of the promoterless *lacZ* gene, generating plasmids p*yscA*, p*lcrG*, p*yscN*, p*yopN*, p*psaA*, p*yopQ*, p*yopJ*, p*yopT* and p*ompC*. Those pRW50 derivatives were then transformed into strain 201 and the *ΔrovA* mutant, respectively. The empty plasmid pRW50 was also introduced into both strains as a negative control for the β-galactosidase activity assay.

For overexpression of RovA, YopE, YopM and YopJ, each of those genes was amplified with the primers listed in Table S1 and then cloned into pET28a, generating pHKR, pHKE, pHKM and pHKJ. The sequences of the expression vectors were verified by DNA sequencing.

### RNA isolation, microarray hybridization and analysis

Strain 201 and the *ΔrovA* mutant were grown in TMH with 2.5 mM calcium at 26°C to an OD_600_ of 1.0. The cultures were diluted 20-fold into fresh TMH without calcium and grown at 26°C until reaching an OD_600_ of about 1.0. For the induction of T3SS, the cultures were then transferred to 37°C for 3 h. Immediately before being harvested, bacterial cultures were mixed with RNAprotect bacterial reagent (Qiagen) to minimize RNA degradation. Total RNA was isolated using a MasterPure RNA purification kit (Epicenter), and the contaminating DNA was removed. RNA quality was monitored by agarose gel electrophoresis, and RNA quantity was determined by spectrophotometry. The gene expression profile of the wild type and the *ΔrovA* strains were analyzed and compared using a *Y. pestis* whole-genome cDNA microarray as described previously [22]. The ratio of mRNA levels was calculated for each gene, and significant changes in gene expression were identified with Significance Analysis of Microarrays (SAM) software as described previously [41]. Only genes with at least a two-fold change in expression were selected for further analysis.

### Gel electrophoresis and Western blotting

For detection of *rovA* expression, strain 201, the Δ*rovA* mutant and *ΔrovA-*pAraRovA were grown in BHI broth at 26°C, and the expression of RovA in *ΔrovA-*pAraRovA was induced by adding arabinose to a final concentration of 0.2∼0.002% in the culture medium. Bacterial cells were collected by centrifugation and weighed before they were resuspended in volumes of SDS-polyacrylamide (SDS-PAGE) gel loading buffer in proportion to the weight of bacterial pellet (300 µl loading buffer per 25 mg). Samples were separated by 15% SDS-PAGE and then transferred to an Immobilon-P membrane (Millipore). The bound proteins were then probed with a polyclonal antibody directed against RovA. The antigen-antibody complexes were visualized with a secondary goat anti-rabbit IgG conjugated to horseradish peroxidase (Sigma).

For analysis of Yop secretion and expression, bacterial strains were grown in TMH medium without calcium at 26°C to an OD_600_ of about 1.0, and then transferred to 37°C for 3 h to induce the expression and secretion of Yops. The culture supernatant and the bacterial cells were separated by centrifugation and the cell pellets were weighed. After addition of 10% (v/v) trichloroacetic acid (TCA), the supernatants were incubated on ice at 4°C overnight to precipitate the proteins. The TCA-precipitated proteins were then collected by centrifugation at 12 000×g for 30 min at 4°C. After the pH was adjusted to 7.0 by adding 5 µl of Tris-HCl buffer (pH 8.9), the precipitated proteins were dissolved in SDS-PAGE loading buffer in proportion to the weight of the bacterial pellet as described above. Proteins from the supernatant and the bacterial pellets were separated and detected using rabbit polyclonal antibodies against YopE, YopJ and YopM, as described above.

### Preparation of polyclonal antibodies against RovA and Yop proteins

BL21λDE3-harboring plasmids pHKR, pHKE, pHKM and pHKJ were grown in LB broth at 37°C and induced with 1 mM IPTG. The cells were resuspended in 50 mM NaH_2_PO_4_, pH 8.0, 300 mM NaCl, 10 mM imidazole, lysed by sonication at 10% power in a Bandelin Sonoplus HD 2200 sonicator (Bandelin Electronic, Berlin, Germany), and the soluble 6×His-tagged RovA, YopE, YopM and YopJ extract was separated from insoluble cell material by centrifugation at 14 000×g. The 6×His-tagged proteins were purified by affinity chromatography on Ni-NTA agarose (Qiagen).

Polyclonal antibodies were generated in rabbits by sequentially injecting 200 µg and 400 µg of purified proteins mixed in Freund's complete adjuvant (Sigma) at two-week intervals. One month later, the rabbits were boosted with a mixture of 800 µg purified proteins with incomplete adjuvant (Sigma). IgG was purified form rabbits' serum by ammonium sulfate precipitation.

### Growth rate determination

Overnight cultures of bacterial strains in TMH medium were diluted 20-fold into fresh TMH, with or without 2.5 mM calcium supplementation, to an OD_600_ of ∼0.1, and allowed to grow at 26°C to the OD_600_ of 0.3, and then transferred to 37°C for incubation until 24 h. The OD_600_ for each culture was monitored every hour by a Bioscreen C (Oy Growth Curves Ab Ltd, Helsinki, Finland).

### Determination of LD_50_

Bacteria for infection were cultured at 26°C in LB broth for 18–20 h. Bacterial cultures were serially 10-fold diluted in phosphate-buffered saline (PBS) to bacterial suspensions of 10 to 10^4^ colony forming units (CFU) per milliliter. Actual numbers of CFU/ml were determined by plating serial dilutions of the cultures on LB agar plates. Six- to eight-week-old female BALB/c mice from the Laboratory Animal Research Center (AMMS) were used in virulence determination, and all animal experiments were conducted in accordance with the Guidelines for the Welfare and Ethics of Laboratory Animals of China. Four groups of six animals were challenged by *s.c.* injection at inguina or *i.v.* injection via the vena caudalis with serial 10-fold dilutions of bacterial cell suspension. Mice were observed daily for 14 days, and the LD_50_ was calculated using the Reed-Muench method [42].

### Electrophoresis mobility shift assays (EMSA)

Primers were designed for amplification of ∼500-600-bp sequences upstream of *lcrG, yscA, lcrF* and *psaE*. EMSA was performed as reported previously [43], [44] using Gel Shift Assay Systems (Promega). In brief, the 5′ ends of PCR products were labeled using [γ-32P] ATP and T4 polynucleotide kinase. DNA binding was performed by mixing binding buffer [20 mM Tris-HCl (pH 8.0), 50 mM KCl, 1 mM DTT, 5% glycerol, 0.05 mg/ml poly-(dI-dC)] with labeled DNA and various concentrations of the purified His-RovA protein and followed by incubation at room temperature for 30 min. The products were separated by 4% (w/v) native PAGE in 0.5×TBE buffer. Radioactive species were detected by autoradiography after exposure to Kodak film at −70°C.

### β -Galactosidase activity assay

*Y. pestis* strain 201 and the *ΔrovA* mutant strains harboring the plasmids p*yscA*, p*lcrG*, p*yscN*, p*yopN*, p*psaA*, p*yopQ*, p*yopJ*, p*yopT* and p*ompC* were grown as described for the DNA microarray analysis. β-Galactosidase activity was measured in cellular extracts using a β-galactosidase enzyme assay system (Promega) according to the manufacturer's instructions.

### Electron microscopy

Bacterial strains were grown in TMH medium with calcium at 26°C to early stationary phase (OD 0.8∼1.0), or grown in TMH medium with/without calcium at 26°C then transferred to 37°C for 3 h when the OD_600_ reached 0.3. Bacterial cells were harvested and fixed in 3% glutaraldehyde in 0.1 M buffer pH 7.4 (Sigma) at 4°C. Thin section transmission electron microscopy was carried out by the Laboratory of Pathology at Inst of Microbiol and Epidemiol, AMMS. The specimens were examined using a Philips Technai 20 TEM (Philips, Netherlands) supported with Mega View II software.

### Membrane permeability assay by CFSE staining

*Y. pestis* strain 201, the *ΔrovA* mutant and the *ΔrovA-*pAraRovA were grown in TMH medium without calcium at 26°C to OD_600_ of 0.8∼1.0. Samples were centrifuged and cell pellets were resuspended in 0.01 M PBS. CFSE (Invitrogen) solution in PBS was added to bacterial suspensions to reach a final concentration of 1 µM, and the mixtures of dye and bacterial cells were incubated at 37°C for 10 min. To stop the staining procedure, samples were centrifuged and the precipitated cells were thoroughly washed twice with PBS, and then resuspended in fresh PBS solution. Bacteria were subjected to analysis using a Becton-Dickinson FACS Caliber flow cytometer.

## Supporting Information

Table S1 Primers used in this study.
(0.03 MB XLS)

Table S2 Genes differently expressed in the rovA mutant as determined by DNA microarray analysis.
(0.04 MB XLS)

Figure S1 Functional classification of RovA regulated genes according to *Y. pestis* CO92 Genome Project.
(0.10 MB JPG)
