# Supplementary figures and images for: Cell Membrane Is Impaired, Accompanied by Enhanced Type III Secretion System Expression in Yersinia pestis Deficient in RovA Regulator
Source: PLoS One. 2010 Sep 17;5(9):e12840. doi: 10.1371/journal.pone.0012840 (PMC2941471; doi:10.1371/journal.pone.0012840)

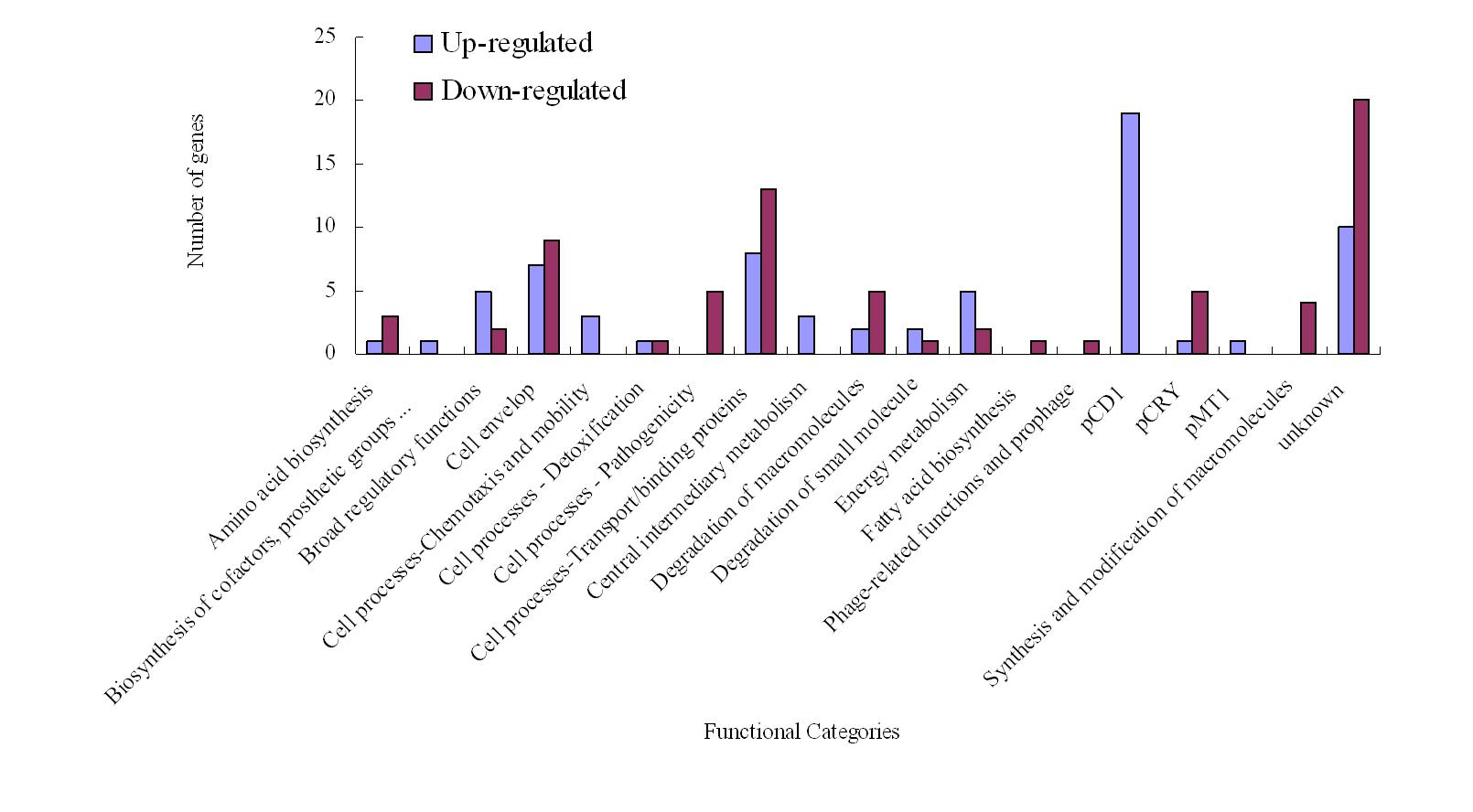

Supplement: Figure S1 — Functional classification of RovA regulated genes according to Y. pestis CO92 Genome Project. (0.10 MB JPG) [file pone.0012840.s003.jpg]
